# Hydrolysis of nicosulfuron under acidic environment caused by oxalate secretion of a novel *Penicillium oxalicum* strain YC-WM1

**DOI:** 10.1038/s41598-017-00228-2

**Published:** 2017-04-05

**Authors:** Weimin Feng, Zheng Wei, Jinlong Song, Qiao Qin, Kaimin Yu, Guochao Li, Jiayu Zhang, Wei Wu, Yanchun Yan

**Affiliations:** 1 Graduate School, Chinese Academy of Agricultural Sciences, Beijing, 100081 China; 2 Insitute of Crop Science/Natonal Key Facility for Crop Gene Resources and Genetic Improvement, Chinese Academy of Agriculture Sciences, Beijing, 100081 China; 3 Chinese Academy of fishery sciences, Beijing, 100141 China

## Abstract

A novel *Penicillium oxalicum* strain YC-WM1, isolated from activated sludge, was found to be capable of completely degrading 100 mg/L of nicosulfuron within six days when incubated in GSM at 33 °C. Nicosulfuron degradation rates were affected by GSM initial pH, nicosulfuron initial concentration, glucose initial concentration, and carbon source. After inoculation, the medium pH was decreased from 7.0 to 4.5 within one day and remained at around 3.5 during the next few days, in which nicosulfuron degraded quickly. Besides, 100 mg/L of nicosulfuron were completely degraded in GSM medium at pH of 3.5 without incubation after 4 days. So, nicosulfuron degradation by YC-WM1 may be acidolysis. Based on HPLC analysis, GSM medium acidification was due to oxalate accumulation instead of lactic acid and oxalate, which was influenced by different carbon sources and had no relationship to nicosulfuron initial concentration. Furthermore, nicosulfuron broke into aminopyrimidine and pyridylsulfonamide as final products and could not be used as nitrogen source and mycelium didn’t increase in GSM medium. Metabolomics results further showed that nicosulfuron degradation was not detected in intracellular. Therefore, oxalate secretion in GSM medium by strain YC-WM1 led to nicosulfuron acidolysis.

## Introduction

Nicosulfuron, a member of the sulfonylurea herbicides used to control broad-leaved weeds and sedge weeds in corn, rice, citrus, vines, and potatoes field, was introduced into China and became the utmost frequently used herbicide on account of its high herbicidal activity at low application rates^1^. The persistence of nicosulfuron in dugout water was longer than sulfosulfuron and rimsulfuron^2^. However, high concentrations of nicosulfuron in industrial wastewater, groundwater, and soil have been detected owing to its strong transferability. The residues of nicosulfuron in soil, surface waters, and some crops have been proved to be a menace to human's health, animal life as well as to soil microbes and have raised increasing concerns about the risk of contamination of nearby aquatic systems or damage to crop rotation.

Sulfonylurea herbicides in natural environment can be removed via chemical hydrolysis^3–5^, microbial metabolism^6–8^, and photocatalytic degradation^9–18^. Microbes play an important role in the degradation of nicosufuron and numerous microbes isolated with the capacity of detoxifying have been proved to be one of the significant means to control environmental pollution. Song *et al*. isolated a fungal strain LZM1 from activated sludge with the capability of degrading nicosulfuron, which may be used in bioremediation of nicosulfuron-contaminated environments. Zhang *et al*. obtained a bacterium *Serratia marcescens* N80 with the degradation rate of 93.6% in 96 hours with nicosulfuron initial concentration at 10 mg/L^19^. Several sulfonylurea herbicide-degrading strains, such as *Serratia sp*. BW30, *Ochrobactrum sp*. LS, *Methylopila sp*. S113, and *Bacillus subtilis* strain YB1 have been reported^20–22^. All kinds of ecological problems related to sulfonylurea herbicides have stimulated efforts to employ biological control agents.

Metabolomics approach not only can provide a comprehensive profile of all the metabolites, but also discover meaningful metabolites in biological systems^23, 24^. The analytical platforms, including gas chromatography–mass spectrometry (GC–MS)^25^, capillary electrophoresis mass spectrometry (CE–MS), and liquid chromatography mass spectrometry (LC–MS) have been applied to microbe, plant, and animal metabolism analysis^26^. GC–MS has been widely used in the fields of functional genomics, metabolomics, and compounds identification because of its high accuracy and good repeatability^27, 28^.

The objective of this study was to isolate a new microorganism species capable of efficiently degrading nicosulfuron. Here, we described the isolation, identification and degradation characteristics of *Penicillium oxalicum sp*. YC-WM1, which secreted oxalate and decreased the environmental pH values leading to nicosulfuron acidolysis. In addition, *Penicillium oxalicum sp*. YC-WM1 exhibited the highest nicosulfuron degradation efficiency compared to the other reported strains.

## Results

### Isolation and identification of YC-WM1

Through the enrichment culture method, a nicosulfuron-degrading fungus was isolated and named strain YC-WM1. YC-WM1 could degrade 100% of the initial nicosulfuron (100 mg/L) in GSM medium within three days (33 °C, inoculum biomass amount, 1.0 g dry wt/L). YC-WM1 was an obligate aerobe when grown on GSM plates. Colonies of YC-WM1 were green with smooth, dense, and powder-like textured surface and its conidia were spherical.

**Figure 1:**
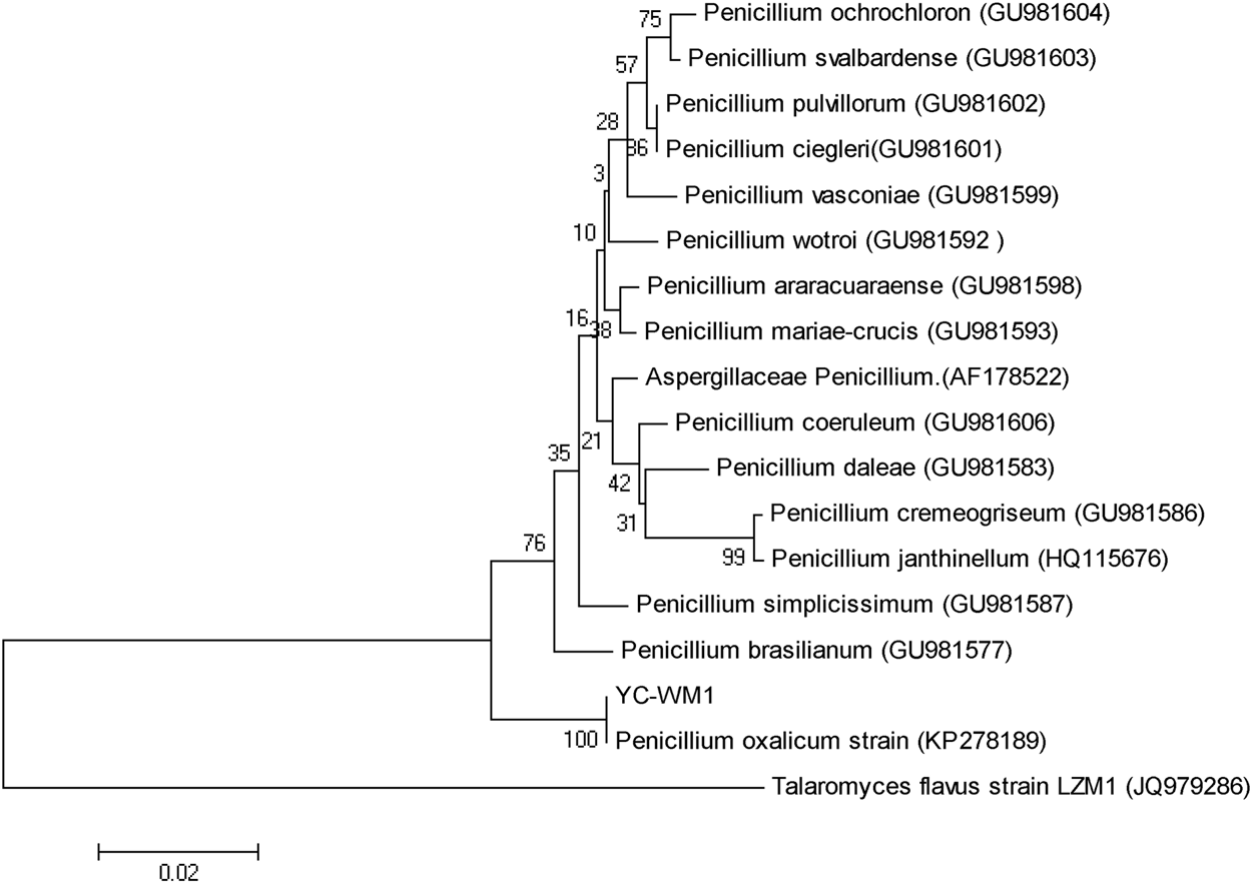
Phylogenetic tree based on ITS sequences of YC-WM1 and relevant strains. The GenBank accession numbers for each microorganism employed in the analysis were shown behind the species name. The scale bar indicated 0.001 substitutions per nucleotide position. Bootstrap values obtained with 1000 resamplings were indicated as percentages at all branches. Talaromyces flavus strain 1 was a fungus reported with the capable of degrading nicosulfuron.

A 561 bp ITS fragment of YC-WM1 was sequenced and phylogenetic analysis (Fig. 1, ITS fragment was offered in supplementary file) was performed based on the ITS sequences which revealed that YC-WM1 was closely related to *Penicillium oxalicum* (100%). According to its morphological characteristics and phylogenetic analysis, YC-WM1 was identified as a *Penicillium oxalicum* strain. So far, this is the first time the *Penicillium oxalicum* species has been reported to be capable of degrading nicosulfuron.

### Degradation of nicosulfuron by YC-WM1

**Figure 2:**
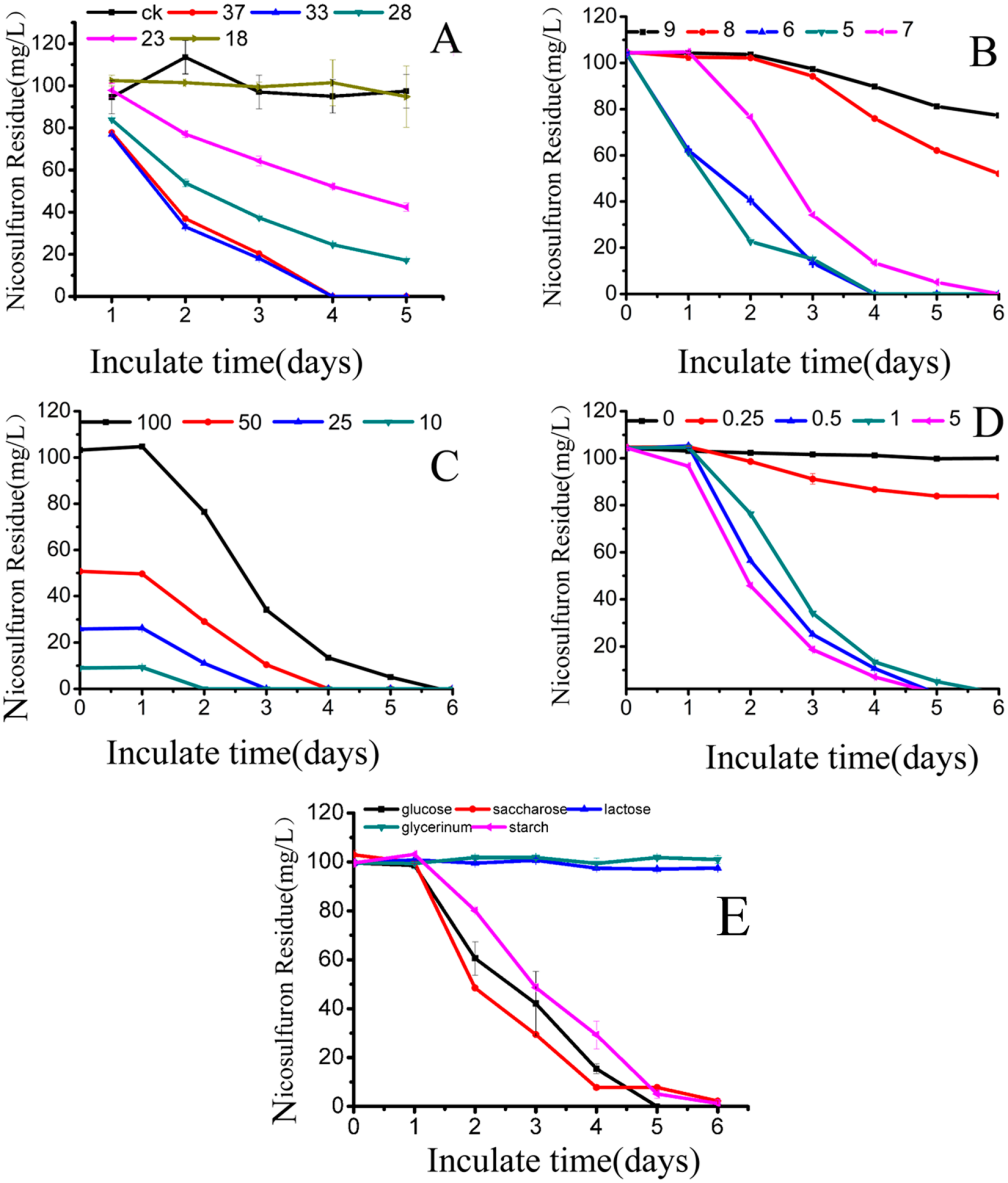
Effect of initial temperature (**A**), pH (**B**), nicosulfuron concentration (**C**), glucose concentration (**D**), and different carbon sources on nicosulfuron degradation by YC-WM1.

The degradation ability of YC-WM1 were studied in GSM medium with nicosulfuron as the sole nitrogen source at different temperatures (18, 23, 28, 33, and 37 °C), pH values (ranging from 5 to 8), initial concentrations of nicosulfuron (100, 50, 25, and 10 mg/L), and carbon sources (glucose, starch, sucrose, lactose, and glycerinum). As shown in Fig. 2A, nicosulfuron couldn’t be detected after 3 days and the degradation rate came to 100% when the temperature was 33 °C and 37 °C (Fig. 2A). So, we chose 33 °C as the optimal temperature for further analysis. Degradation of nicosulfuron under different initial pH conditions was explored to analyze its stability. The results (Fig. 2B) showed that when the initial pH values were higher than 7.0, degradation rates of nicosulfuron gradually decreased as the pH values increased. When the initial pH values were 5.0–7.0, the degradation rates of nicosulfuron were all 100% after 120 hours, indicating that YC-WM1 can adapt to neutral and weakly acidic environment (Fig. 2B).

Under pH 7.0, YC-WM1 was incubated in GSM medium at 33 °C for 6 days with nicosulfuron as the sole nitrogen source at concentrations of 10, 25, 50, and 100 mg/L. For any group, the degradation rates of nicosulfuron were all 100% after 6 days, and it took about 2, 3, 4, and 6 days for different groups to degradate the nicosulfuron, which indicated that YC-WM1 possessed high potential values of application. The more nicosulfuron added, the more time it would take for complete degradation (Fig. 2C).

The effect of initial concentrations of glucose was then studied on the performance of nicosulfuron degradation by YC-WM1. YC-WM1 was cultured under 33 °C and pH 7.0 for 6 days in GSM medium with different concentrations of glucose ranging from 0.25 g/L to 5 g/L while 100 mg/L of nicosulfuron was added. We found that when the initial glucose concentrations were 5, 1, and 0.5 g/L, 100 mg/L of nicosulfuron were totally degraded after 6 days. However, the degradation rate was only 19% when the glucose concentration was 0.25 g/L. Nicosulfuron was hardly degraded without glucose in the GSM medium (Fig. 2D). Our study showed that the nicosulfuron degradability of YC-WM1 is significantly affected by glucose content in the GSM medium.

To analyze the importance of carbon sources to nicosulfuron degradation by YC-WM1, YC-WM1 was cultured in the GSM medium with different carbon sources, including glucose, sucrose, starch, lactose, and glycerinum. 100 mg/L nicosulfuron was supplemented. We found that the degradation rates were 100%, 98%, and 97% when the carbon sources were glucose, sucrose, and starch, respectively. However, nicosulfuron in GSM medium were hardly degraded when the carbon sources were lactose and glycerinum (Fig. 2E). Our study showed that the nicosulfuron degradability of YC-WM1 is significantly affected by carbon source.

#### The nicosulfuron degradation rates by strain YC-WM1 varied along with the change of pH values in the GSM medium

**Figure 3:**
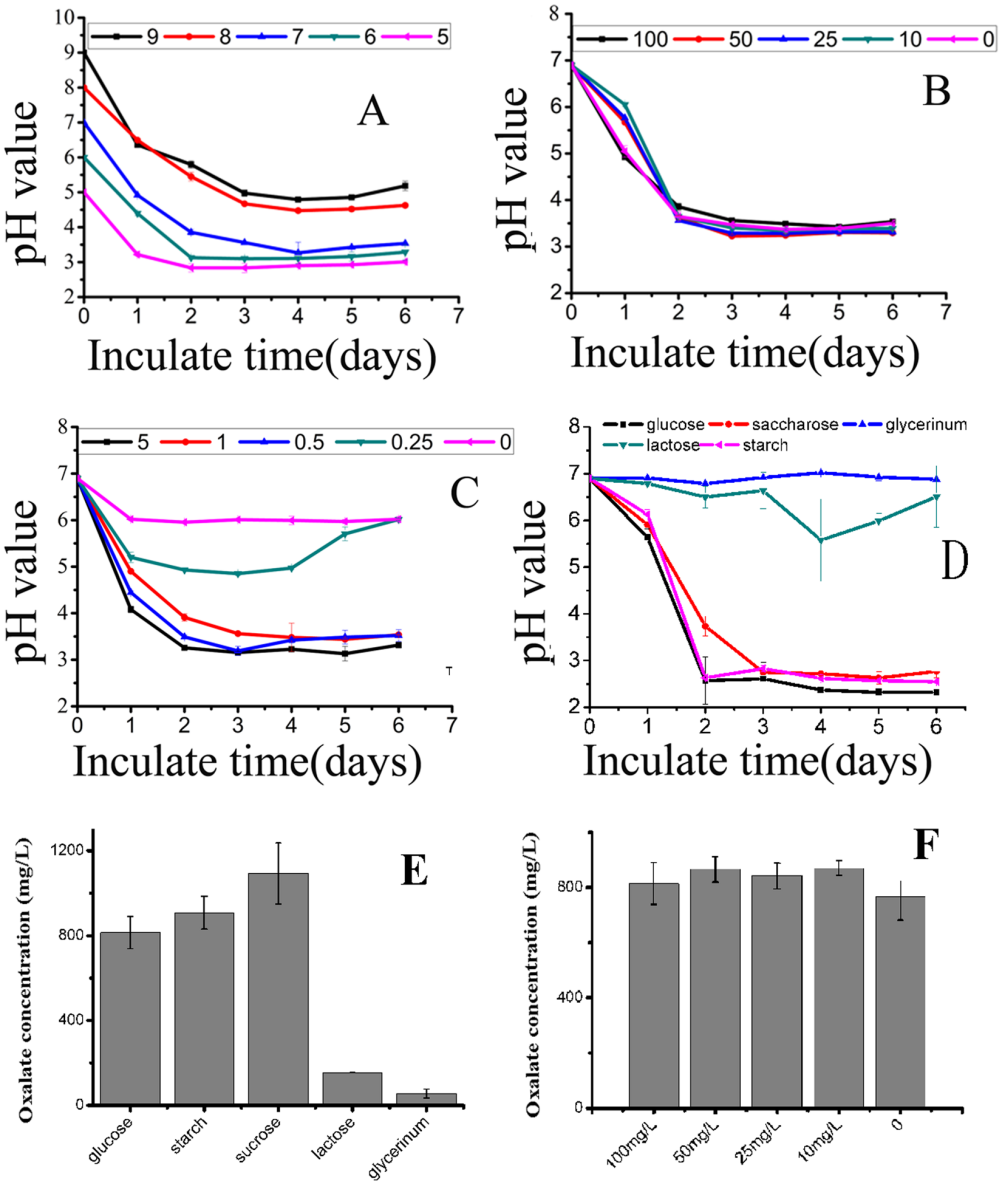
GSM medium pH value changes with various initial pH (**A**), initial nicosulfuron concentration (**B**), glucose initial concentration (**C**) and different carbon sources (**D**). Oxalic production changes (48 hours incubation) with initial nicosulfuron concentration (**E**) and carbon sources (**F**).

The dynamic change of pH values in the GSM medium was monitored during the course of nicosulfuron degradation by YC-WM1. Our results showed that the pH values declined from 7 to 3.5 after inoculation for 2 days and little difference was detected afterwards. As shown in Fig. 3A, the dynamic change of pH values in GSM medium was correlated with the initial pH values. When the initial pH values were 5, 6, and 7, it dropped below 4 while 4.8 and 5.1 with initial pH values of 8 and 9, respectively. But, no significant difference was observed among groups with different initial concentrations of nicosulfuron as mentioned above (Fig. 3B). This indicated that dynamic change of pH values in GSM medium had nothing to do with nicosulfuron initial concentrations.

An interesting finding in Fig. 3C was that the dynamic change of pH values was closely related with initial concentrations of glucose. When the initial concentrations of glucose were 5, 1, and 0.5 g/L, pH values were decreased to 3.4, 3.5, and 3.5 after 2 days, respectively. Simultaneously, pH values were much higher at any given time when little or no glucose was added into the GSM medium. Next, we manually adjusted the pH values of the solution to 7 every 24 hours, nicosulfuron was not degraded by YC-WM1 after incubation for 5 days. It was proposed that the degradation of nicosulfuron was caused by hydrolysis under acidic conditions due to metabolism of glucose by strain YC-WM1. In other words, the degradation of nicosulfuron was actually caused by the combined effects of microorganisms on acid secretion and chemical hydrolysis.

The dynamic change of pH values was found to be closely related to carbon sources of the GSM medium (Fig. 3D). The pH values of GSM medium were decreased to 2.57 and 2.63 after 2 days when the carbon sources were glucose and starch, respectively. When the carbon sources was sucrose, the GMS medium pH values dropped to 3.74 and 2.75 after 2 and 3 days, respectively, at a relatively slower rate. The variation of pH values could be hardly observed when the carbon sources were glycerinum and lactose. Glycerinum and lactose weren’t common carbon sources for bacteria and fungi on research of pesticide degradation. The hydrolysis products of starch are glucose while sucrose equally break into glucose and fructose. Before starch and sucrose were used as direct carbon source, it took some steps for starch and sucrose being decomposed into monosaccharide (glucose and fructose), and then the monosaccharide participated in glycolysis. However, these steps are enzymatic reactions which are very fast. Moreover, one molecule of sucrose and starch could be deposed into two and many monosaccharide molecules, respectively. So, the decrease rates of pH values were not obviously different when starch and sucrose were chosen as carbon sources compared to glucose. Starch-Iodine Color Reaction showed that starch was exhausted in two days (Fig. S4).

Then we found that 100 mg/L of nicosulfuron were completely degraded in 4 days when added into GSM medium at pH of 3.5 without inoculation, which was consistent with the results stated above. So nicosulfuron is unstable under acid environment (especially under pH 5) and the mechanism of nicosulfuron degradation by YC-WM1 may be acidolysis.

#### Relationship between mycelia growth and nicosulfuron degradation

The effect of different carbons and different initial concentrations of nicosulfuron on mycelia growth were studied. However, little variation on dry weight of mycelia were observed during the whole incubation while the pH values were decreased dramatically and nicosulfuron were degraded rapidly (Figs S5 and S6). Therefore, nicosulfuron degradation by YC-WM1 was due to acidolysis rather than enzyme catalysis.

### Hypothesis of mechanisms on nicosulfuron degradation by strain YC-WM1

To define the possible mechanism of nicosulfuron degradation by strain YC-WM1. The HPLC was applied to confirm the cleavage of the sulfonylurea group. We observed that the peak area designated for nicosulfuron was decreasing over time. Aminopyrimidine and pyridysulfonamide^29^ were detected in the GSM medium after nicosulfuron was degraded by YC-WM1 (Figs S1 and S2). To check whether aminopyrimidine and pyridylsulfonamide could be metabolized by YC-WM1, the corresponding chemical standard substances were added into GSM and MSM inoculated with YC-WM1. We found that the concentrations of these two substances didn’t decrease after being inoculated for 5 days. So, nicosulfuron turned into aminopyrimidine and pyridylsulfonamide as final products and could not be used as nitrogen source, which prevent YC-WM1 from proliferation.

In the meantime, oxalate were accumulated in the GSM medium before and after nicosulfuron degradation by YC-WM1 (Fig. S3). There was a strong correlation between carbons source and oxalate yield 48 h after inoculation. In detail, when the carbon sources were glucose, starch, and sucrose, the concentration of oxalate in the GSM medium reached to 814, 908, and 1093 mg/L, respectively. The pH values in the GSM medium were around 3.5. However, the concentration of oxalate were only 154 and 55 mg/L with the carbon sources of lactose and glycerinum and the pH values were above 6 (Fig. 3E). No relationship was found between nicosulfuron initial concentrations and oxalate production on the second day. The pH values in the GSM medium were all around 3.5 (Fig. 3F). In addition, some other organic acids, such as lactic acid and acetic acid, were not detected in GSM medium described above (Fig. S3). Therefore, the decrease of pH values in the GSM medium by strain YC-WM1 was due to oxalate accumulation, which led to nicosulfuron acidolysis.

### Metabolic analysis of strain YC-WM1

**Figure 4:**
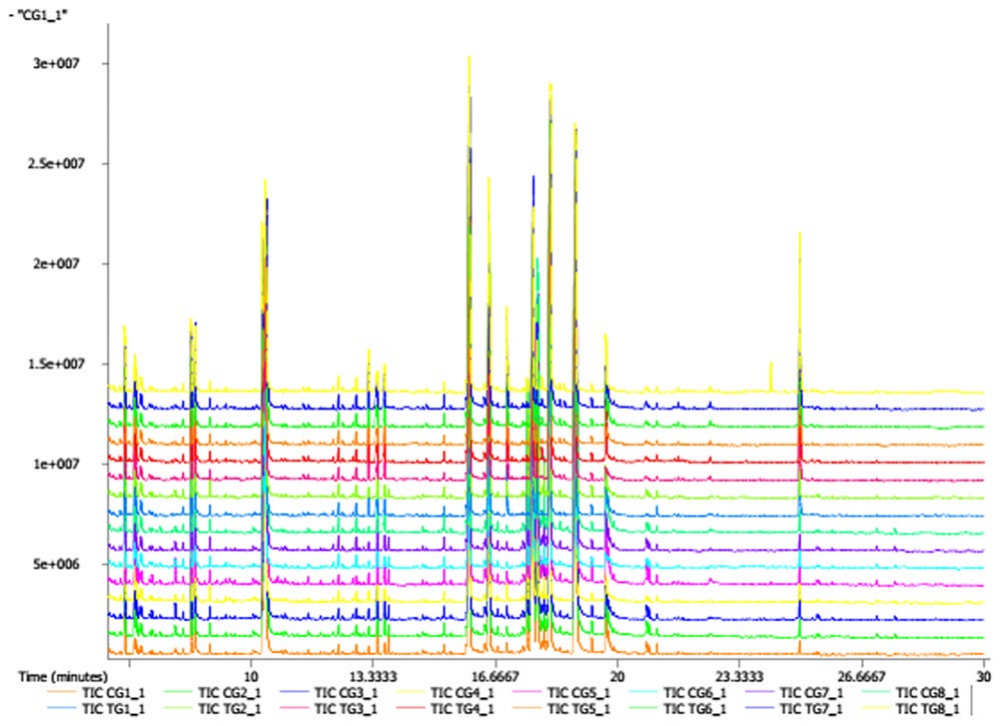
GC-TOF/MS TIC chromatograms of YC-WM1 with and without nicosulfuron exposure (TIC TG and TIC CG).

The GC-TOF/MS TIC chromatograms of strain YC-WM1 with or without nicosulfuron exposure were shown in Fig. 4. There were various differences in the shape and quantity of peaks between different treatments, with unique peaks in each biofluid.

**Figure 5:**
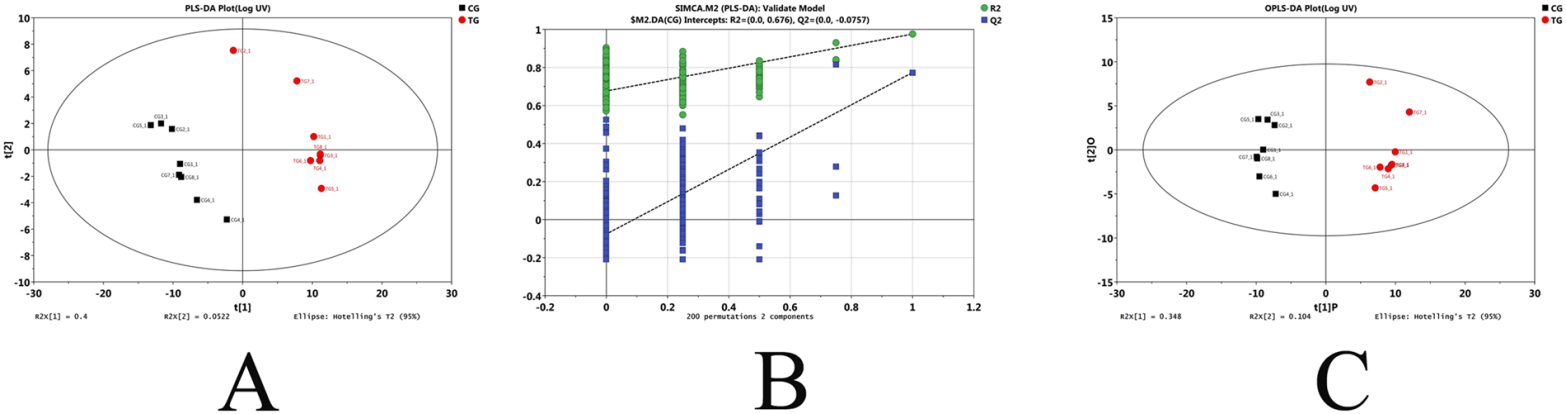
PCA score plots of PLS-DA (**A**), validation plots of PLS-DA (**B**) and PCA score plot PLS-DA Plot (**C**) derived from the GC-TOF/MS metabolite profiles of YC-WM1 in the culture with (TG) and without nicosulfuron (CG).

251 peaks were detected in the strain YC-WM1 and 249 annotated metabolites were identified by GC–MS. 249 annotated metabolites contained about 34 amino acids, 96 organic acids, 42 carbohydrates, 16 alcohols, 13 amines, 10 fatty acids, 46 various organic class compounds, and 1 inorganic compound. Compared to the control groups (CGs), concentrations of 67 metabolites increased in the nicosulfuron treated groups (TGs), while 93 decreased (p < 0.05). The similarity and diversity of samples with different treatments were determined by the method of principal component analysis (PCA) where parallel samples were clustered (Fig. 5). The results showed that metabolites were noticeably separated between the TGs and CGs.

**Figure 6:**
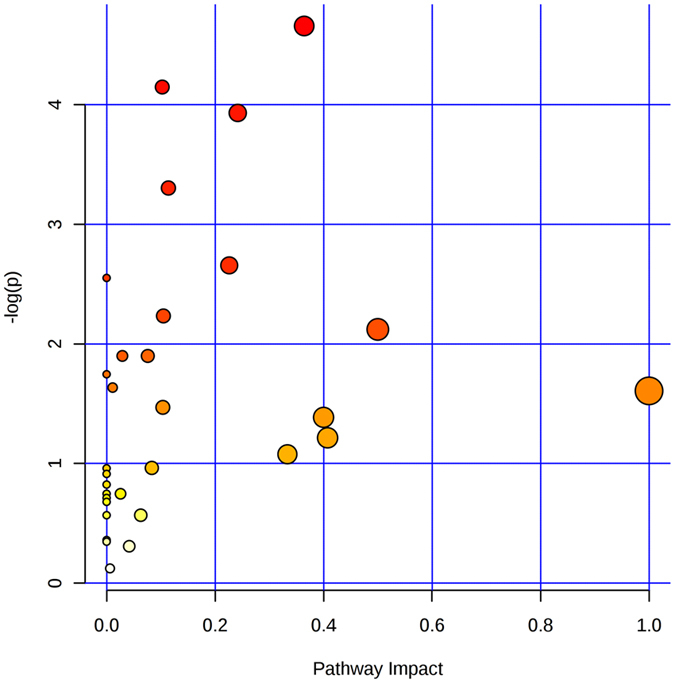
Metabolome view maps of the common metabolites identified in YC-WM1 with and without nicosulfuron. The x-axis represents the pathway impact, and y-axis represents the pathway enrichment. Larger sizes and darker colors represent higher pathway enrichment and higher pathway impact values.

As shown in Fig. 6, 30 metabolism pathways (p < 0.05, the pathway impact values were higher than 0.1), including 160 metabolites with significant difference between the TGs and CGs, were enriched. However, only 20 of them were up-regulated and the other 10 were down-regulated, as shown in Table 1.

**Table 1:** Metabolic Pathways enrichment from the Significantly Different Metabolites between TG and CG.

	Enriched pathway	Hits	Impact	Change
1	Butanoate metabolism	3	0.57	down
2	Glycine, serine and threonine metabolism	3	0.40	down
3	Arginine and proline metabolism	5	0.30	down
4	Pentose phosphate pathway	3	0.25	down
5	Alanine, aspartate and glutamate metabolism	4	0.25	down
6	Nitrogen metabolism	2	0.24	down
7	Glycerophospholipid metabolism	2	0.22	down
8	Methane metabolism	2	0.17	down
9	Cysteine and methionine metabolism	2	0.15	down
10	Phenylalanine, tyrosine and tryptophan biosynthesis	3	0.10	down
11	beta-Alanine metabolism	2	1.00	up
12	Citrate cycle (TCA cycle)	5	0.37	up
13	Galactose metabolism	2	0.33	up
14	Glyoxylate and dicarboxylate metabolism	3	0.25	up
15	Amino sugar and nucleotide sugar metabolism	3	0.20	up
16	Arginine and proline metabolism	5	0.17	up
17	Inositol phosphate metabolism	2	0.16	up
18	Starch and sucrose metabolism	3	0.13	up
19	Lysine biosynthesis	2	0.13	up
20	Pyruvate metabolism	2	0.12	up

The pathways mentioned above were cross-linked together. As for TCA cycle (KEGG pathway map 00020), nicosulfuron treatment significantly enhanced the production of L-malic acid, citric acid, oxalacetic acid, acidalpha-ketoglutaric acid, fumaric acid, and succinic acid (P < 0.05). Fumaric and L-malic acids were important intermediates in energy generation. The increase of intermediates in glycolysis (KEGG pathway map 00010) might indicate that energy related metabolism was enhanced in response to nicosulfuron treatment. The obvious increase of fructose levels in TGs would have a great impact on regulation of the glycolytic metabolism and even the entire glycolysis pathway. The enhanced energy metabolism was considered as a typical adaptive response under nicosulfuron stress, which is a ubiquitous mechanism existing among animals, plants, and microbe. Therefore, the enhancement of energy metabolism might contribute to nicosulfuron-induced resistant response. Besides, some organic substances, including 6-phosphogluconic acid, D-glyceric acid, trehalose, sucrose, gluconic acid, ribose-5-phosphate, gluconic acid, D-ribose, and 6-phosphogluconic acid related to pentose phosphate pathway (KEGG pathway map 00030), were down-regulated in the TGs (P < 0.05).

In regards to amino acid metabolism, most pathways were down-regulated under nicosulfuron treatment, including alanine, aspartate, and glutamate metabolism (KEGG pathway map 00030), arginine and proline metabolism (KEGG pathway map 00330), butanoate metabolism (KEGG pathway map 00650), arginine and proline metabolism (KEGG pathway map 00330) and lysine degradation (KEGG pathway map 00310) pathways. Only beta-Alanine metabolism (KEGG pathway map 00410) pathway was up-regulated.

In this study, although pyruvate metabolism (KEGG pathway map00620) pathway was up-regulated in TGs, pyruvate and its downstream molecules were not detected. Malic acid, oxaloacetic acid, and citrate, with higher concentrations in TGs, actually did not participate or had little involvement in pyruvate metabolism.

On the contrary, nicosulfuron, aminopyrimidine, and pyridylsulfonamide were not detected among the various metabolites, which further proved that nicosulfuron hydrolysis occurred in the extracellular circumstance of the fungal cells. Propanoate metabolism (KEGG pathway map00640) was promoted in TGs while the concentration of oxalate was of no significant difference between TGs and CGs.

Higher concentrations of phenylethylamine in TGs indicated that nicosulfuron may act as a stressor for YC-WM^30^. Glycerophospholipids, as glycerol-based phospholipids, were main components of biological membranes and play an important role in the generation of both extracellular and intracellular signals. Glycerophospholipid metabolism was suppressed in TGs, which may guarantee the process of cellular transport.

## Discussion

Microbes play an important role in degradation of herbicides in natural environment. Numerous microbial groups that can degrade or inactivate these chemicals have been identified. Degradation efficiency of tribenuron methyl (TBM) by *Pseudomonas sp*. strain NyZ42 was about 80% with initial concentration of 200 mg/L within 4 days^31^. Some other microbes, capable of degrading metsulfuron-methy, have been isolated^32, 33^. A group of microbes that can degrade chlorimuron-ethyl have also been identified^32, 34, 35^. In addition, *Serratia marcescens* N80 with nicosulfuron as the sole nitrogen source, could degradate 93.6% nicosulfuron with initial concentration of 10 mg/L in 96 hours. *Serratia marcescens* N80 also had degradation function on some other sulfonylurea herbicides, including ethametsulfuron, tribenuron-methyl, metsulfuron-methyl, chlorimuron-ethyl, and rimsulfuron^19^. In our study, a nicosulfuron-hydrolyzing strain YC-WM1 was isolated from nicosulfuron contaminated sludge and identified as *Penicillium oxalicum*.

At present, degradating mechanism of sulfonylureas is still not very clear. Two patterns of sulfonylureas degradation have been reported, pure biological process^5^ and microbial acidohydrolysis^22^. As for biological degradation pattern, only three relevant proteins purified from *Bacillus subtilis* YB1 with the capacity of nicosulfuron degradation were identified, namely manganese ABC transporter, vegetative catalase 1, and acetoin dehydrogenase E1^21^. According to some reports, the degradation of sulfonylureas herbicides was accounted for acidolysis^5, 22, 36^. Sulfonylurea herbicides are unstable under acidic conditions due to low isoelectric point. For instance, strain BW30 was capable of converting glucose (or other carbon compounds in soil) into short-chain fatty acids, including oxalic and lactic acids. These short-chain fatty acids then attacked the sulfonylurea bridge and finally resulted in the breakdown of TBM molecules^37^. In our study, glucose metabolism was essential to nicosulfuron degradation by *Penicillium oxalicum* YC-WM1. Firstly, we measured the amount of oxalate in the GSM culture inoculated with YC-WM1 for 3 days and accorded the pH value. The oxalate concentration was 832 mg/L and the pH value was 2.57. Then, we added equal amount of oxalate to the GSM medium without inoculation of YC-WM1 and detected its pH value. It was interesting that the pH value was very close to 2.57. So, the decrease of pH values during the process of nicosulfuron degradation by *Penicillium oxalicum* YC-WM1 was due to the accumulation of oxalate produced by glucose metabolism. As nicosulfuron is unstable under acidic conditions and its degradation rates by YC-WM1 were closely related to pH variations and initial concentrations of glucose, so the degrading mechanism of nicosulfuron by YC-WM1 belonged to microbial acidolysis. Lately, *Alcaligenes faecalis* ZWS11 was recorded with the character of degrading nicosulfuron. However, Alcaligenes faecalis ZWS11 did not utilize nicosulfuron as carbon sources or nitrogen source because the GSM medium they described contained carbo sources (glucose) and nitrogen source (NH_4_NO_3_)^2^. So, the degradation mechanism of nicosulfuron by ZWS11 should be further comfirmed.

Metabolites trigger cellular reactions in different pathways that mediate and perform multiple cellular functions. Metabolite profiling changes in biological functions or phenotypes are common in response to genetic or environmental stimulation^29, 38, 39^. GC-TOF/MS-based metabonomics were used in conjunction with multivariate statistics to examine the metabolite alteration of *Penicillium oxalicum* YC-WM1 induced by nicosulfuron exposure. PLS-DA and OPLS-DA analysis of metabolism showed great differences between the TGs and CGs, which indicated that nicosulfuron exposure affected the metabolism of *Penicillium oxalicum* YC-WM1 to a certain extent. We not only compared the differential levels of metabolites between TGs and CGs, but also pinpointed the corresponding pathways^40, 41^. Based on metabolic pathway analysis, 20 significant metabolic pathways have been identified from the TGs compared to the CGs.

The pentose phosphate pathway, a biochemical pathway parallel to glycolysis, can generate NADPH and 5-carbon sugars and involves oxidation of glucose. However, its primary role is anabolic metabolism rather than catabolic action (such as fatty-acid synthesis and assimilation of inorganic nitrogen)^11, 39, 42^. The reasons for down-regulation of the pentose phosphate pathway may be due to the lack of N source and the enhanced energy metabolic activity under nicosulfuron stress.

Oxalic acid, a metabolic end product for plants and fungi, has three chemical natures: proton and electron source and a strong metal chelator^26^. In GSM medium inoculated with *Penicillium oxalicum* YC-WM1, large amounts of oxalate were accumulated resulting in decrease of pH values, which led to acidolysis of nicosulfuron. In fungi, the biosynthesis of oxalate occurs in cell via the glyoxylate cycle or the tricarboxylic acid cycle^43^. The substances related to glyoxylate cycle and tricarboxylic acid cycle were all detected except glyoxylate in intracellular, including citrate, isocitrate, succinate, fumarate, malate, oxaloacetate. At the final step, oxalate is produced either by oxidation of glyoxylate to oxalate or cleavage of oxaloacetate to oxalate and acetate^44^.

**Figure 7:**
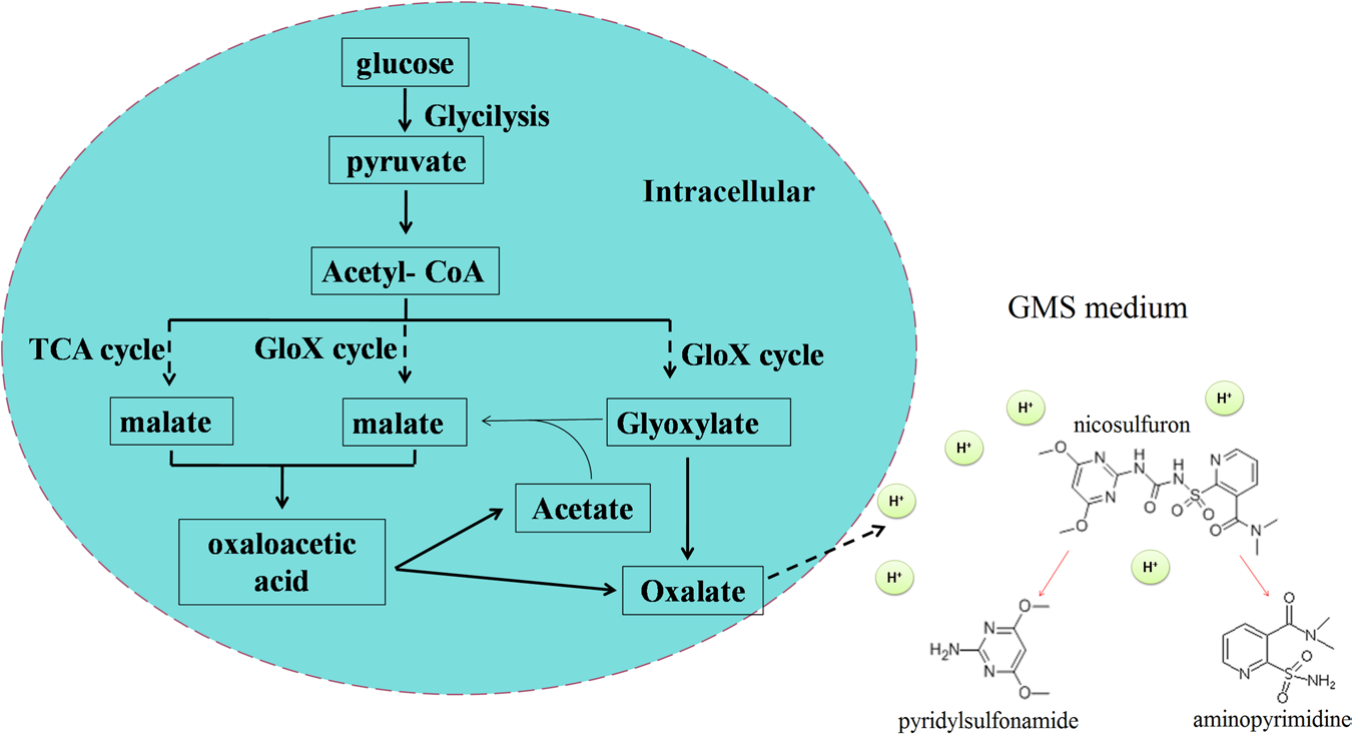
A sketch of nicosulfuron hydrolysis by *Penicillium oxalicum* YC-WM1.

Upon these findings, we proposed a biological adaptation model (Fig. 7). Amount of oxalate was generated and secreted into the environment which led to the acidolysis of nicosulfuron since nicosulfuron is unstable under acidic conditions.

## Methods

### Chemicals and Media

Nicosulfuron (analytical grade, >99%) was purchased from Chem. Service Co, USA. High- Performance liquid chromatatography (HPLC)-grade acetonitrile was obtained from sigma Aldrich USA. All other reagents used were of analytical grade. The following media were used to isolate nicosulfuron-degrading strains: enrichment culture containing 10 g/L tryptone, 5 g/L yeast extraction, and 10 g/L NaCl; mineral salt culture (MSM) containing 2 g/L (NH_4_)_2_SO_4_, 0.2 g/L MgSO_4_•7H_2_O, 0.01 g/L CaCl_2_•2H_2_O, 0.001 g/L FeSO_4_•H_2_O, 1.5 mg/L Na_2_HPO_4_•12H_2_O, and 1.5 g/L KH_2_PO_4_; glucose salt culture (GSM) containing 1 g/L glucose, 1.5 g/L K_2_HPO_4_•12H_2_O, 1 g/L KH_2_PO_4_, 0.5 g/L NaCl and potato glucose culture (PDA) (Hope Bio-Technology Co., Ltd., China.).

### Enrichment, isolation, and screening of nicosulfuron-degrading strains

Nicosulfuron-contaminated sludge was collected from Shandong Huayang Science and Technology Co., Ltd., a pesticide manufacturer in Shandong, China. Ten milliliters of activated sludge were transferred into 250 mL Erlenmeyer flasks containing 100 mL of sterilized enrichment culture with 100 mg/L nicosulfuron. The enrichment culture was incubated in a rotary shaker (100 rpm) at 30 °C in the dark^45^. After 7 days, cultures were serially diluted and plated on GSM agar plates supplemented with 600 mg/L nicosulfuron. The plates were incubated at 30 °C, 100 rpm for 5 days, and a single fungal colony was selected and pure colonies were obtained by restreaking for three times. The ability of isolates to degrade nicosulfuron was determined by HPLC, using an Agilent 1200 system equipped with a C_18_ column and operated with gradient elution of a solvent mixture (1 ml/min) of acetonitrile (20%) and distilled water containing 0.05% acetic acid (80%).

### Identification and characterization of nicosulfuron-degrading strains

The isolate with the capacity of nicosulfuron degradation was selected for further analysis, while the characterization and identification were based on its morphological and internal transcribed spacer (ITS) sequence analysis. The ITS sequence was amplified by PCR with the universal primers: ITS1 (5′-TCCGTAGGTGAACCTGCGG-3′) and ITS4 (5′- TCCTCCGCTTATTGATATGC-3′). The PCR was carried out as following: 10 min of denaturation at 95 °C, followed by 29 cycles of 95 °C for 1 min, 55 °C for 30S, and 72 °C for 1 min. An extending of 10 min at 72 °C followed the last cycle. The amplified ITS products were detected by 1.0% agarose gel electrophoresis and used as the template for direct sequencing of ITS by Sangon Biotech Co., Ltd. (Shanghai, China). Phylogenetic tree was constructed using the neighbor-joining method and the maximum composite likelihood model plugged in MEGA 5.10 software^46^.

### Degradation experiments of nicosulfuron by YC-WM1

The following important parameters that significantly influenced nicosulfuron biodegradation rates and their optimized ranges were chosen as independent variables in this experiment: incubation temperature (18, 23, 28, 33, and 37 °C, inoculum biomass amount, 1.0 g dry wt/L), pH values (5.0, 6.0, 7.0, 8.0, and 9.0, inoculum biomass amount, 0.5 g dry wt/L) and different carbon sources (glucose, sucrose, lactose, glycerinum and starch). YC-WM1 was inoculated in GSM containing 100 mg/L nicosulfuron as the sole nitrogen source, which was used to detect the residues in the liquid medium every day. Biodegradation experiments of nicosulfuron by YC-WM1 at different initial concentrations (25, 50 and 100 mg/L) were also conducted under the optimum growing conditions to determine the optimum initial concentration (inoculum biomass amount, 0.5 g dry wt/L) (Chen *et al*., 2011). Medium without inoculation of strain YC-WM1 served as a control. The influence of different concentrations of glucose (0.25, 0.5, 1.0 and 5.0 g/L) was also analyzed with the same inoculum biomass amount (0.5 g dry wt/L). In the end, The influence of different carbon source (glucose, starch, sucrose, lactose, and glycerinum, 1.0 g/L) was also analyzed with the same inoculum biomass amount (0.5 g dry wt/L), The residual concentrations of nicosulfuron was determined by HPLC (Agilent 1200, USA). At the same time, the pH values were monitored with HI 2221 calibration check pH/ORP meter every 24 hours. The effect of different carbon sources and initial concentration of nicosulfuron on dry weight of mycelium were monitored every 24 hours.

### Identification of metabolites in GSM culture

After being incubated for 2 days in 100 mL liquid medium containing 100 mg/L nicosulfuron, several metabolites, such as aminopyrimidine, pyridyl sulfonamide, lactic acid and so on, were detected by an Agilent 1200 HPLC.

#### Metabonomics analysis of YC-WM1 related with nicosulfuron degradation

Extraction of metabolites in fungal YC-WM1.

YC-MW1 was inoculated in GSM liquid medium containing 100 mg/L nicosulfuron for 2.5 days when 50% of the initial nicosulfuron was hydrolyzed. The mixture was transferred to 50 mL tubes and centrifuged to pellet cells at 6000 rpm. The supernatant was discarded; the pellet was washed thrice and resuspended in PBS.

A certain amount of yellow basket-shaped mycelium samples was placed into 2 mL tubes. 50 μL of L-2-Chlorophenylalanine (0.1 mg/mL stock solution in ddH_2_O), as an internal standard, and 0.4 mL of the extraction. To homogenize the samples, steels balls were placed into the sample tubes then homogenized in ball mill for 5 minutes at 70 Hz. The samples were then centrifuged at 12000 rpm for 15 minutes at 4 °C. 0.35 mL of the supernatant were transferred into a fresh 2 mL GC/MS glass vial.

#### Derivation of metabolites extracted from fungal YC-WM1

The samples were dried in a vacuum concentrator at low temperature. 80 μL of methoxyamination reagent (20 mg/mL in pyridine) was added to the samples and kept for 2 hours at 37 °C. 0.1 mL of BSTFA regent (1% TMCS, v/v) was then added to the sample aliquots and kept for 1 hour at 70 °C. The samples were left to cool to room temperature and were later used for GC-MS analysis.

### Detection of metabolites extracted from fungal YC-WM1

GC/TOF MS analysis was performed using an Agilent 7890 gas chromatograph system coupled with a Pegasus HT time-of-flight mass spectrometer. The system utilized a DB-5MScapillary column coated with 5% diphenyl cross-linked with 95% dimethylpolysiloxane (30 m × 250 μm inner diameter, 0.25 μm film thickness, J&W Scientific, Folsom, CA, USA). 1 μL aliquot of the analyte was injected in splitless mode. Helium was used as the carrier gas. At rate of 1 ml/min through the column and the front inlet purge flow was 3 ml/min. The initial temperature was kept at 50 °C for 1 minute, then raised to 330 °C at a rate of 10 °C/min, then kept for 5 minutes at 330 °C. The injection, transfer line, and ion source temperatures were 280, 280, and 220 °C, respectively. The energy was −70 eV in electron impact mode. The mass spectrometry data were acquired in full-scan mode with the m/z range of 85–600 at a rate of 20 spectra/s after a solvent delay of 360 s.

#### Data analysis

In this study, missing values of raw data were filled up using half of the minimum values across samples, and 248 peaks were detected. So, 248 metabolites were left through interquartile range denoising method. In addition, internal standard normalization method was employed in this data analysis. The resulting three-dimensional data involving the peak number, sample name, and normalized peak area were fed to SIMCA-P 11.5 software (Umetrics, Umea, Sweden) for principal component analysis (PCA), partial least squares discriminant analysis (PLS-DA) and orthogonal projections to latent structures-discriminant analysis (OPLS). PCA analysis showed the distribution of the original data. In order to obtain a higher level of group separation and get a better understanding of variables responsible for classification, a PLS-DA analysis was applied. Afterwards, the parameters for the classification from the software were R2Y = 0.976 and Q2Y = 0.773 which were stable and good to fitness and prediction. A 7-fold cross-validation was used to estimate the robustness and the predictive ability of our model. Such permutation test was proceeded in order to further validate the model. The R2 and Q2 intercepted values were after 200 permutations. The low value of Q2 intercept indicate the robustness of the models, and thus show a low risk of over fitting and reliable. Based on OPLS analysis, a loading plot was constructed, which showed the contribution of variables to difference between the experimental group and the control group. It also showed the significant variables which were situated far from the origin but the loading plot was complex because of many variables. To refine this analysis, the first principal component of variable importance projection (VIP) was obtained. VIP values exceeding 1.0 were first selected as changed metabolites. In step 2, the remaining variables were then assessed by Student's T test (T-test, P < 0.05), and variables were discarded between two groups. In addition, commercial databases, including KEGG (http://www.genome.jp/kegg/) and NIST (http://www.nist.gov/index.html) were utilized to search for metabolites. The heat map was established by R project. Metaboanalyst (www.metaboanalyst.ca/) was used for pathway construction by employing the rice metabolic pathway databases as reference for global test algorithm.

## Electronic supplementary material

Supplementary file
