# Supplementary material for: Hydrolysis of nicosulfuron under acidic environment caused by oxalate secretion of a novel Penicillium oxalicum strain YC-WM1
Source: Sci Rep. 2017 Apr 5;7:647. doi: 10.1038/s41598-017-00228-2 (PMC5428040; doi:10.1038/s41598-017-00228-2)
Supplement: Supplementary file 1 — Supplementary file [file 41598_2017_228_MOESM1_ESM.doc]

**Hydrolysis of nicosulfuron under acidic environment caused by oxalate secretion of a novel *Penicillium oxalicum* strain YC-WM1**

Weimin Feng,^1,a^, Zheng Wei,^1,2^ Jinlong Song,^1,3^ Qiao Qin,^2^ Kaimin Yu,^1^ Guochao Li,^1^ Jiayu Zhang,^1^ Wei Wu, ^1^ and Yanchun Yan^1.*^

^1^Graduate School, Chinese Academy of Agricultural Sciences, Beijing 100081,

China

^2^Insitute of Crop Science/Natonal Key Facility for Crop Gene Resources and Genetic Improvement,  Chinese Academy of Agriculture Sciences, Beijing 100081, China.

^3^Chinese Academy of fishery sciences, Beijing 100141, China.

*corresponding author:

Yanchun Yan (E-mails: [Yanyanchun2012@qq.com](mailto:Yanyanchun2012@qq.com) and yanyanchun@caas.cn)

**The ITS sequence of YC-WM-1**

GATGGGTGCTACCTGATCGAGGTCACCTGGTTAAGATTGATGGTGTTCGCCGGCGGGCGCCGGCCGGGCCTACAGAGCGGGTGACGAAGCCCCATACGCTCGAGGACCGGACGCGGTGCCGCCGCTGCCTTTCGGGCCCGCCCCCCGGAAGCGGGGGGCGAGAGCCCAACACACAAGCCGTGCTTGAGGGCAGCAATGACGCTCGGACAGGCATGCCCCCCGGAATACCAGGGGGCGCAATGTGCGTTCAAAGACTCGATGATTCACTGAATTCTGCAATTCACATTACTTATCGCATTTCGCTGCGTTCTTCATCGATGCCGGAACCAAGAGATCCGTTGTTGAAAGTTTTAACTGATTTAGTCAAGTACTCAGACTGCAATCTTCAGACAAGAGTTCGTTTGTGTGTCTTCGGCGGGCGCGGGCCCGGGGGCGGATGCCCCCCGGCGGCCGTGAGGCGGGCCCGCCGAAGCAACAAGGTACGATAAACACGGGTGGGAGGTTGGACCCAGAGGGCCCTCACTCGGTAATGATCCTTCCGCAGGTCCCCTAACGGAAGGA


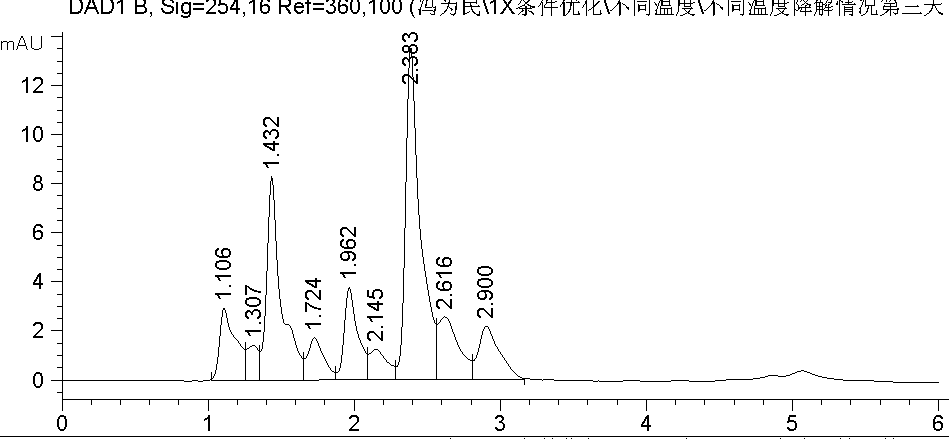


**Figure S1 Detection of pyridylsulfonamide in nicosulfuron metabolites of YC-WM1 by HPLC. pyridylsulfonamide retention time was 1.432.**


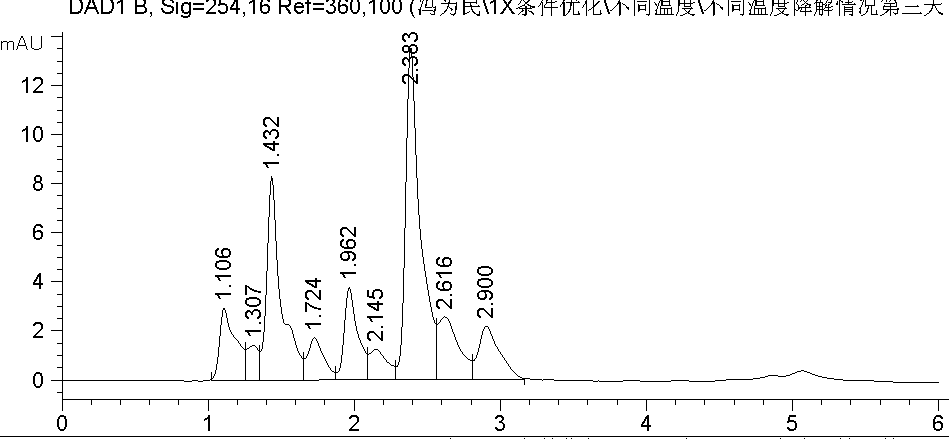


**Figure S2 Detection of aminopyrimidine in nicosulfuron metabolites of YC-WM1 by HPLC. Aminopyrimidine retention time was 2.383.**


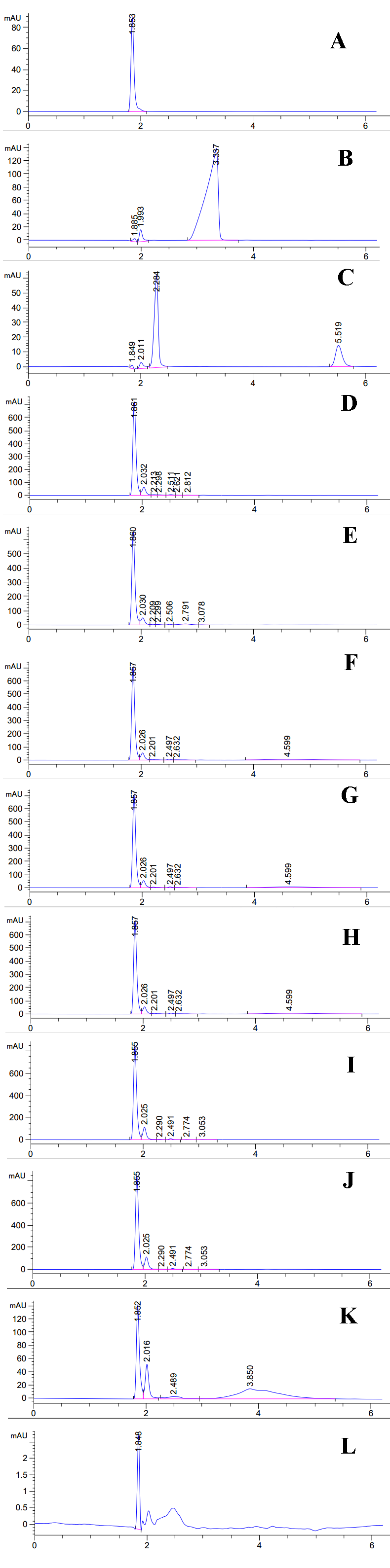


Figure S3 **The HPLC detection of oxalate concentration the second day in GSM medium with the carbon source and different nicosulfuron initial concentration.**

The high performance liquid chromatography of standard substance of oxalate(A), acetic acid(B), lactic acid(C), The high performance liquid chromatography of the oxalate concentration in GSM medium inoculation of YC-WM1 with nicosulfuron initial concentration of 100, 50, 25, 10, 0mg/L(D-H), The high performance liquid chromatography of the oxalate concentration in GSM medium with the carbon source change to stach, sucrose, lactose, and glycerinum inoculating of YC-WM1 with 100 mg/L nicosulfuron (I-L), the mobile phase of HPLC is 2% PO_3_H_2_NH_4_. flow velocity of mobile phase is 0.7 ml/min


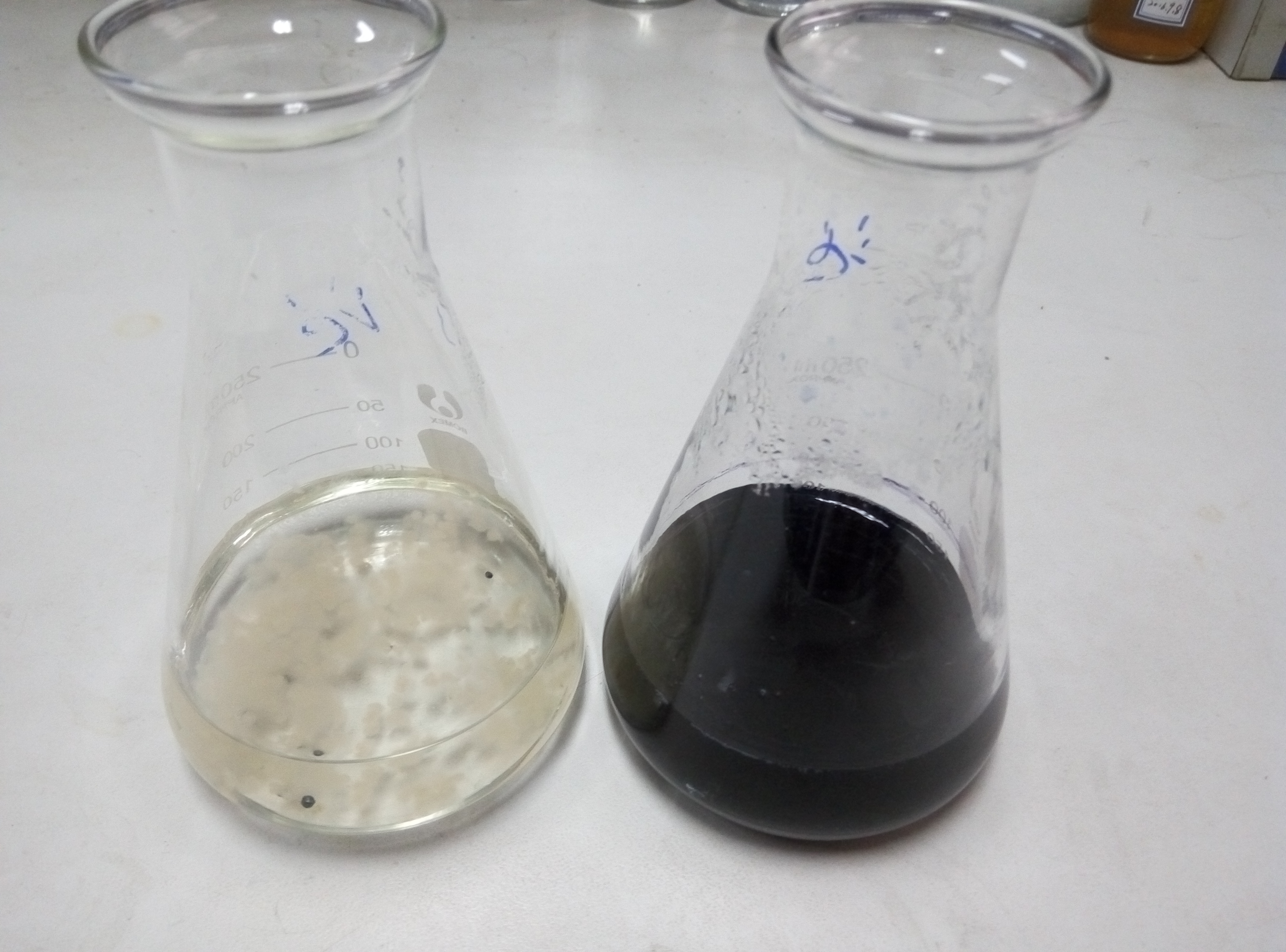


A

B

**Figure S4 Starch-Iodine Color Reaction in GSM with the carbon source incubating for 2 days with inoculating mycelia (A) and without inoculating (B).**


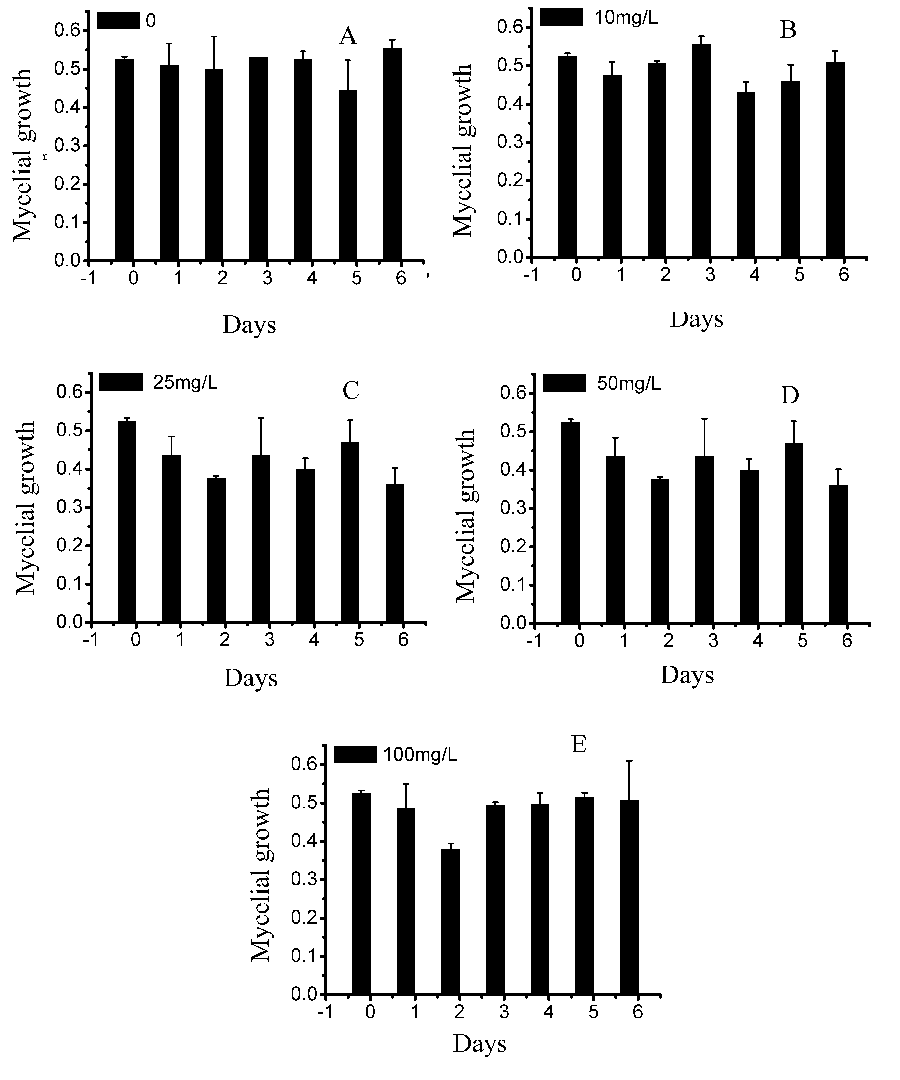


**Figure S5 The effects on mycelial growth of YC-WM1 in GMS medium with the nicosulfuron concentration of 0(A), 10(B), 25(C), 50(D), and 100(E) mg/L.**

**
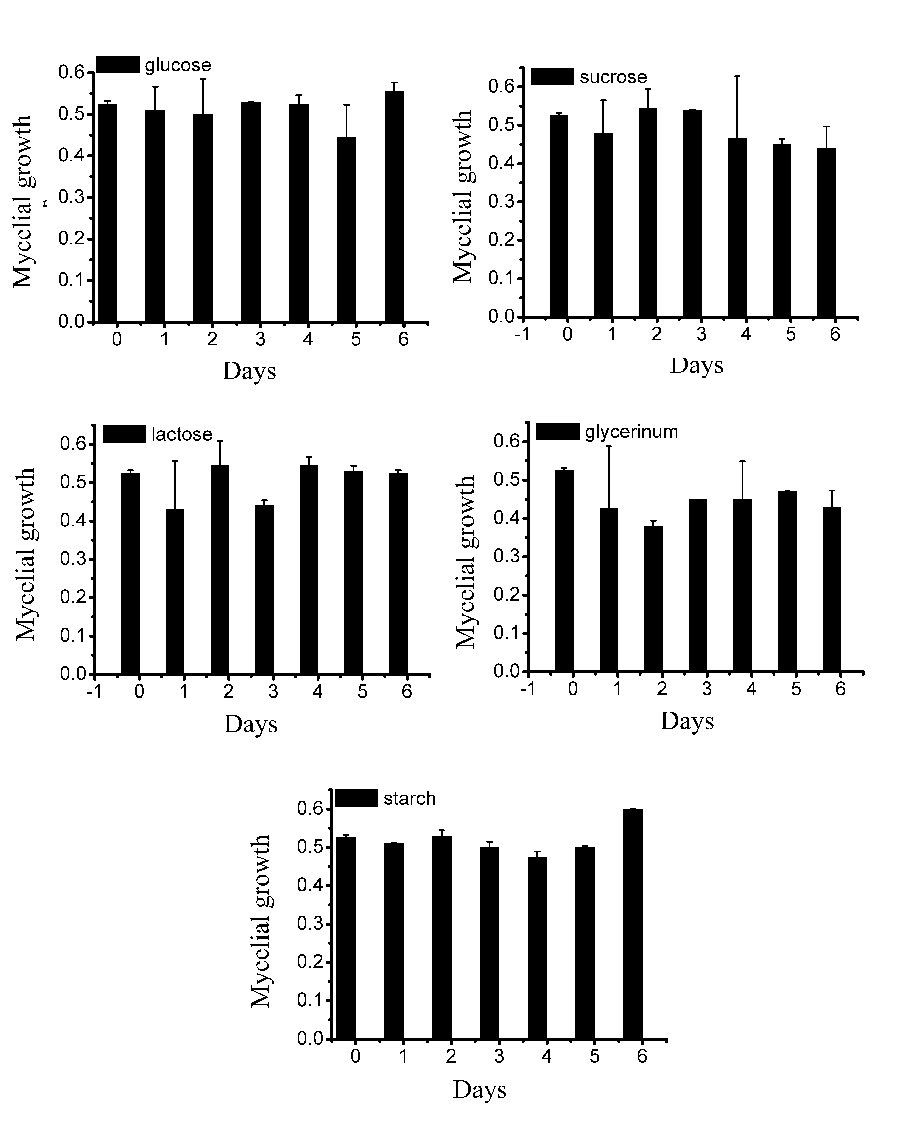
**

**Figure S6 The effects on mycelial growth of YC-WM1 in GMS medium with the carbon sources of glucose(A), surose(B) , lactose(C), glycerinum(D), and starch(E).**
